# Antiarrhythmic Agents and the Risk of Malignant Neoplasm of Liver and Intrahepatic Bile Ducts

**DOI:** 10.1371/journal.pone.0116960

**Published:** 2015-01-15

**Authors:** Yun-Ping Lim, Cheng-Li Lin, Yen-Ning Lin, Wei-Chih Ma, Wei-Cheng Chen, Dong-Zong Hung, Chia-Hung Kao

**Affiliations:** 1 Department of Pharmacy, College of Pharmacy, China Medical University, Taichung, Taiwan; 2 Department of Emergency, Toxicology Center, China Medical University Hospital, Taichung, Taiwan; 3 Management Office for Health Data, China Medical University Hospital, Taichung, Taiwan; 4 Department of Pharmacy, National Cheng Kung University Hospital Dou-Liou Branch, Yunlin, Taiwan; 5 Graduate Institute of Clinical Medical Science and School of Medicine, College of Medicine, China Medical University, Taichung, Taiwan; 6 Department of Nuclear Medicine and PET Center, China Medical University Hospital, Taichung, Taiwan; National Health Research Institutes, TAIWAN

## Abstract

**Background:**

The objective of this study was to determine the association between the use of antiarrhythmic agents and the risk of malignant neoplasm of liver and intrahepatic bile ducts (MNLIHD).

**Methods:**

We used the research database of the Taiwan National Health Insurance Program to conduct a population-based, case-control study. We identified 9944 patients with antiarrhythmic history who were first diagnosed as having MNLIHD between 2005 and 2010. We identified an additional 19,497 patients with antiarrhythmic history in the same period who did not develop MNLIHD and were frequency-matched using age, sex, and index year to form a control group. Five commercially available antiarrhythmic agents, amiodarone, mexiletine, propafenone, quinidine, and procainamide, were analyzed.

**Results:**

The adjusted odds ratio (OR) of MNLIHD was 1.60 (95% confidence interval [CI], 1.45–1.77) for amiodarone users versus nonamiodarone users. In subgroup analysis, amiodarone use was significantly associated with an increased risk of MNLIHD with an adjusted OR of 18.0 (95% CI, 15.7–20.5) for patients with comorbidities compared to an OR of 2.43 (95% CI, 1.92–3.06) for those without comorbidities. After adjustment for age, sex, statins, anti-diabetes medications, non-steroidal antiinflammatory drugs, propafenone use, quinidine use, and comorbidities, the ORs were 1.49, 1.66, and 1.79 for MNLIHD associated with annual mean defined daily doses of ≤30, 31–145, and >145, respectively.

**Conclusions:**

The results of the present study indicated that amiodarone might be associated with the development of MNLIHD in a dose-dependent manner, particularly among patients with comorbidities.

## Introduction

The use of antiarrhythmic medication is recommended in routine clinical practice guidelines as the first-line management of atrial fibrillation (AF) [1]. Antiarrhythmic drugs are administered with the aim of reducing the period and incidence of AF, the need for hospitalization, and the incidence of AF-related mortality. However, numerous limitations have been reported for these drugs, including proarrhythmic and noncardiovascular toxicities, and, more frequently, only modest antiarrhythmic efficacy [2]. Despite these limitations, antiarrhythmics are still widely prescribed for the management of symptomatic AF. Several case reports and reviews have addressed the potential adverse effects associated with these agents, including liver toxicity [3–6], hepatic dysfunction [7, 8], steatohepatitis [9], liver injury [10], and intrahepatic cholestatic jaundice [11]. However, few systemic analyses have been conducted in this setting, and little evidence of an association between the use of antiarrhythmic agents and the risk of developing malignant neoplasm of liver and intrahepatic bile ducts (MNLIHD) has been reported.

A large-scale retrospective cohort study conducted in Taiwan revealed a borderline significant increase in cancer mortality among patients assigned to aamiodarone group [12]. Based on a review of the literature, only one relevant longitudinal cohort study has been reported, and no large-scale case-control study to date has addressed the association of MNLIHD with the use of antiarrhythmic drugs in Taiwan. Among MNLIHD, hepatocellular carcinoma (HCC) remained the second leading cause of cancer deaths after lung cancer in Taiwan in 2012, with a mortality rate of 34.9 per 100,000 persons (18.6% of the total cases) [13], and it is therefore critical to address this problem. Accordingly, we conducted a population-based case-control study by using data from the National Health Insurance (NHI) program of Taiwan to evaluate the relationship between the use of antiarrhythmic drugs and the risk of MNLIHD.

## Methods

The NHI Program was established in Taiwan in 1995. It has enrolled up to 99% of the Taiwanese population and contracted with 97% of all medical providers [14]. The National Health Research Institutes (NHRI) is responsible for managing the insurance claims data reported to the Bureau of Health Insurance. For research purpose, the NHRI compiles all medical claims in the NHI program and releases the database annually to the public. In the NHI program, insurants who suffer from certain major diseases, such as malignancies or autoimmunine diseases, and those who require transplants, can apply for a catastrophic illness certificate. Application for a catastrophic illness certificate for malignancies requires cytological or pathological evidence supporting the diagnosis. The NHI database of catastrophic illness integrates multiple NHI databases to provide comprehensive utilization and enrollment information for all patients with severe diseases who obtained copayment exemption from the NHI program. Diagnostics were coded with the International Classification of Disease, Ninth Revision, Clinical Modification (ICD-9-CM).

**Figure 1:**
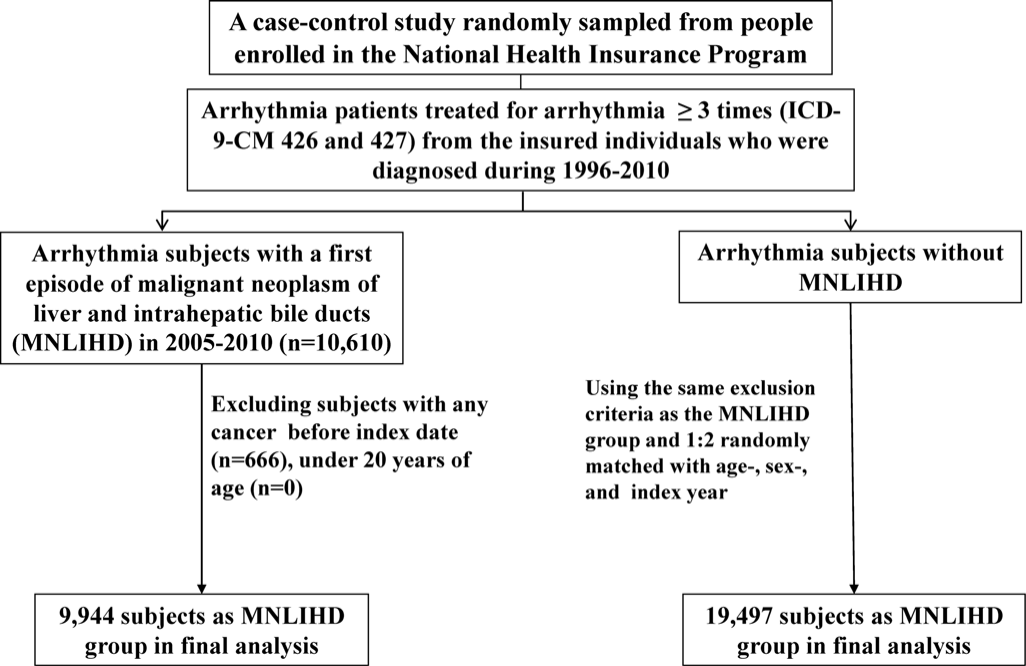
Flow chart of participant recruitment in this study.

Fig. 1 illustrated the sampling scheme in the study. From the Registry of Catastrophic Illness and NHI program databases, we identified arrhythmia (ICD-9-CM codes 426–427) patients newly diagnosed with MNLIHD (ICD-9-CM code 155) in the period from 2005 to 2010 as a case group. Patients who were younger than 20 years of age were excluded. The date of application for MNLIHD was defined as the index date. For each of the MNLIHD patients, we randomly selected 2 arrhythmia patients without MNLIHD from the same period, using the same exclusion criteria, and frequency-matched the case group by using sex and age (5 y a group). A total of 9944 patients with MNLIHD and 19,497 patients without MNLIHD were included in this study.

We considered major comorbidities and medications as covariates, such as diabetes (ICD-9-CM code 250), chronic liver disease and cirrhosis (ICD-9-CM codes 571), hepatitis B virus (HBV) infection (ICD-9-CM codes V02.61, 070.20, 070.22, 070.30, 070.32), hepatitis C virus (HCV) infection (ICD-9-CM codes V02.62, 070.41, 070.44, 070.51, 070.54), alcoholism (ICD-9-CM codes 291, 303, 305.00, 305.01, 305.02, 305.03, 790.3,V11.3), medications of statins, anti-diabetics, and non-steroidal antiinflammatory drugs (NSAIDs) at baseline.

Five commercially available antiarrhythmic drugs in Taiwan were analyzed: amiodarone, mexiletine, propafenone, quinidine, and procainamide. Medication use records were retrieved from ambulatory and inpatient claims data. According to total supply in days and quantity of amiodarone, we calculated the annual mean defined daily dose (DDD) of amiodarone for amiodarone users (ATC C01BD01). For amiodarone, the annual mean DDD was partitioned into 3 levels based on dose tertile.

Sex; age (20–39 y, 40–64y, 65–74y, ≥75 y); medication history of amiodarone, mexiletine, propafenone, quinidine, and procainamide; and comorbidities were compared between the MNLIHD patients and control patients by using a chi-square test. We used a *t* test for continuous variables. A univariate and multivariate logistic regression model was used to calculate the odds ratio (OR) and 95% confidence interval (CI) for factors associated with the risk of MNLIHD. Only confound variables found significantly in the univariable model were further included in the multivariable model. The multivariate analysis was performed to adjust for possible confounders (including medication of propafenone, quinidine, and comorbidities of diabetes, chronic liver disease and cirrhosis, HBV infection, HCV infection, and alcoholism, and medications of statins, anti-diabetics, and NSAIDs). All analyses were performed using SAS statistical software for Windows (Version 9.2; SAS Institute, Inc., Cary, NC, USA), and the significance level was set at.05.

### Ethics Statement

The NHIRD encrypts patient personal information to protect privacy and provides researchers with anonymous identification numbers associated with relevant claim information, including patients’ sex, dates of birth, medical services utilized, and prescriptions. Patient consent is not required for accessing the NHIRD. This study was approved by the Institutional Review Board of China Medical University (CMU-REC-101–012). Our IRB specifically waived the consent requirement.

## Results

The baseline characteristic profiles of the patients are summarized in Table 1. A total of 29,441 arrhythmia patients aged ≥20 years were enrolled in this study. Of the MNLIHD cases, 60.5% were men and 72.8% were ≥65 years of age (Table 1). The mean ages of the MNLIHD patients and non- MNLIHD control patients were 70.6 (±10.9) and 70.3 (±11.2) years, respectively. The proportion of medication history of amiodarone, propafenone, and quinidine was significantly higher for the MNLIHD patients than for the non- MNLIHD control patients. MNLIHD patients were more likely than control patients to exhibit baseline comorbidities (30.7% for diabetes, 91.5% for chronic liver disease and cirrhosis, 34.0% for HBV infection, 42.9% for HCV infection, and 3.63% for alcoholism) and history of medications (40.8% for anti-diabetics and 99.1% for NSAIDs). Compared with the MNLIHD group, the control group was more likely taking statin (31.4% vs 21.0%).

**Table 1:** Baseline Characteristics between Malignant Neoplasm of Liver and Intrahepatic Bile Ducts (MNLIHD) and Non-MNLIHD Group.

	**Non-MNLIHD N = 19,497**		**MNLIHD N = 9944**		
	**N**	**%**	**N**	**%**	P value*
*Gender*					.20
Women	7848	40.3	3926	39.5	
Men	11649	59.8	6018	60.5	
*Age group (year)*					.79
20–39	154	0.79	77	0.77	
40–64	5262	27.0	2631	26.5	
65–74	6812	34.9	3486	35.1	
≥75	7269	37.3	3750	37.7	
Mean (SD) (year) *	70.3	11.2	70.6	10.9	.02
*Medications*					
Amiodarone	2060	10.6	1445	14.5	<.001
Mexiletine	1350	6.92	736	7.40	.13
Propafenone	1218	6.25	707	7.11	<.001
Quinidine	105	0.54	92	0.93	<.001
Procainamide	119	0.61	59	0.59	.86
Statins	6113	31.4	2092	21.0	<0.001
Anti-diabetics	5249	26.9	4058	40.8	<0.001
NSAIDs	19173	98.3	9858	99.1	<0.001
*Baseline comorbidities*					
Diabetes	4118	21.1	3055	30.7	<.001
Chronic liver disease and cirrhosis	10478	53.7	9101	91.5	<.001
HBV infection	730	3.74	3382	34.0	<.001
HCV infection	593	3.04	4262	42.9	<.001
Alcoholism	304	1.56	361	3.63	<.001

*Chi-square test and *t* test comparing subjects with and without MNLIHD. Data are presented as the number of subjects in each group, with percentages given in parentheses.

Table 2 lists the crude and adjusted ORs of MNLIHD risk by arrhythmic drugs use and comorbidities. In the univariate logistic analysis, among the antiarrhythmic drugs we observed only amiodarone that was risk factors of MNLIHD (*P*<.001). Compared with non-amiodarone users, after adjusting for age, sex, use of antiarrhythmic drugs (propafenone and quinidine), and comorbidities, the OR of MNLIHD risk was 1.60-fold (95% CI, 1.45–1.77) higher for amiodarone users. Based on the multivariate analysis, chronic liver disease and cirrhosis (adjusted OR = 3.73, 95% CI, 3.42–4.07), HBV infection (adjusted OR = 15.2, 95% CI, 13.8–16.7), HCV infection (adjusted OR = 20.4, 95% CI, 18.5–22.5), alcoholism (adjusted OR = 2.04, 95% CI, 1.66–2.50), and anti-diabetics (adjusted OR = 2.03, 95% CI, 1.85–2.23) were associated with an increased risk of MNLIHD. However, patients with taking statins (adjusted OR = 0.62, 95% CI = 0.58–0.67) demonstrated a significant association with decreased MNLIHD risk.

**Table 2:** Odds Ratios (OR) and 95% Confidence Intervals (CI) of MNLIHD Associated with Amiodarone and Covariates.

	**Crude**		**Adjusted^†^**	
**Variable**	**OR**	**(95%CI)**	**OR**	**(95%CI)**
*Medications*				
Amiodarone	1.44	(1.34, 1.55)***	1.60	(1.45, 1.77)***
Mexiletine	1.07	(0.98, 1.18)	-	-
Propafenone	1.15	(1.04, 1.26)***	0.97	(0.85, 1.11)
Quinidine	1.73	(1.30, 2.28)***	1.32	(0.91, 1.93)
Procainamide	0.97	(0.71, 1.33)	-	-
Statins	0.58	(0.55, 0.62)***	0.62	(0.58, 0.67)***
Anti-diabetics	1.87	(1.78,1.96)	2.03	(1.85, 2.23)***
NSAIDs	1.94	(1.53, 2.46)***	1.26	(0.92, 1.73)
*Baseline comorbidities*				
Diabetes	1.66	(1.57, 1.75)***	1.05	(0.95, 1.16)
Chronic liver disease and cirrhosis	9.29	(8.61, 10.0)***	3.73	(3.42, 4.07)***
HBV infection	13.3	(12.2, 14.4)***	15.2	(13.8, 16.7)***
HCV infection	23.9	(21.8, 26.2)***	20.4	(18.5, 22.5)***
Alcoholism	2.38	(2.04, 2.78)***	2.04	(1.66, 2.50)***

^†^Adjusted for statins, anti-diabetics, NSAIDS, antiarrhythmic drugs of propafenone, quinidine, comorbidities of diabetes, chronic liver disease, cirrhosis, HBV infection, HCV infection, and alcoholism

*P<.05

**P<.01

***P<.001.

Table 3 illustrates the interaction effect of antiarrhythmic drugs and comorbidities on MNLIHD. Compared with non-amiodarone users without any comorbidities, patients with comorbidities were associated with an increased risk of MNLIHD (adjusted OR = 12.6, 95% CI, 11.3–14.1), followed by patients using amiodarone (adjusted OR = 2.43, 95% CI, 1.92–3.06) and amiodarone users with comorbidities (adjusted OR = 18.0, 95% CI, 15.7–20.5; interaction *P* <.001). Interactions between amiodarone and HBV (adjusted OR = 18.6, 95% CI = 14.4–24.0; interaction *P* = .30) and HCV (adjusted OR = 25.9, 95% CI = 20.1–33.5; interaction *P* = .02) were also associated with increased MNLIHD risk.

**Table 3:** Interaction Effect between Medications and Comorbidities on Risk of MNLIHD.

**Variable**		**OR(95% CI)^†^**	***P value^&^***
*Amiodarone*	*Comorbidity^‡^*		<.001
No	No	As a reference	
No	Yes	12.6 (11.3, 14.1)***	
Yes	No	2.43 (1.92, 3.06)***	
Yes	Yes	18.0 (15.7, 20.5)***	
*Amiodarone*	*HBV*		0.30
No	No	As a reference	
No	Yes	14.0(12.7, 15.3)***	
Yes	No	1.48(1.36, 1.61)***	
Yes	Yes	18.6(14.4, 24.0)***	
*Amiodarone*	*HCV*		0.02
No	No	As a reference	
No	Yes	23.6(21.4, 26.0)***	
Yes	No	1.52(1.39, 1.66)***	
Yes	Yes	25.9(20.1, 33.5)***	
*Mexiletine*	*Comorbidity^‡^*		.29
No	No	As a reference	
No	Yes	11.3 (10.2, 12.9)***	
Yes	No	0.88 (0.58, 1.32)	
Yes	Yes	12.1 (10.5, 13.9)***	
*Propafenone*	*Comorbidity^‡^*		.93
No	No	As a reference	
No	Yes	11.4 (10.4, 12.7)***	
Yes	No	1.18 (0.82, 1.70)	
Yes	Yes	13.5 (11.7, 15.6)***	
*Quinidine*	*Comorbidity^‡^*		.72
No	No	As a reference	
No	Yes	11.4 (10.4, 12.6)***	
Yes	No	1.96 (0.69, 5.62)	
Yes	Yes	17.9 (12.9, 25.0)***	
*Procainamide*	*Comorbidity^‡^*		.48
No	No	As a reference	
No	Yes	11.4 (10.3, 12.6)***	
Yes	No	0.62 (0.15, 2.55)	
Yes	Yes	12.2 (8.43, 17.6)***	

^†^Adjusted for statins, anti-diabetics, and NSAIDS.

^&^ *P* value for interaction.

^‡^Patients with any of the comorbidities (diabetes, chronic liver disease, cirrhosis, HBV infection, HCV infection, and alcoholism) were classified as the comorbidity group.

Furthermore, when estimating risk of MNLIHD based on cumulative DDD for amiodarone use, the amiodarone users were also associated with a higher risk of MNLIHD (Table 4). Compared with the non-amiodarone users, the MNLIHD risk was higher in patients who were administered >145 annual mean DDD of amiodarone (adjusted OR = 1.79, 95% CI, 1.50–2.15), followed by those administered 31–145 annual mean DDD of amiodarone (adjusted OR = 1.66, 95% CI, 1.38–1.99), and those administered ≤30 annual mean DDD of amiodarone (adjusted OR = 1.49, 95% CI, 1.30–1.70). Furthermore, we analyzed the association between MNLIHD and the time differences among the last amiodarone use and index date. Compared with non-amiodarone users, MNLIHD patients with amiodarone use within one year prior to index date were at the greatest risk (adjusted OR = 2.34, 95% CI, 2.05–2.67) and two year prior to index date of developing MNLIHD (adjusted OR = 1.22, 95% CI, 1.06–1.42) (Table 5).

**Table 4:** Odds Ratio (OR) and 95% Confidence Intervals (CI) of MNLIHD Associated with Annual Mean Daily Defined Dose (DDD) Use of Amiodarone.

	**Case/control N**	**Crude OR (95% CI)**	**Adjusted OR ^†^(95% CI)**
**Non-use of Amiodarone**	**8499/17437**	**1.00 (reference)**	**1.00 (reference)**
*Amiodarone*			
≤30 DDD	714/1729	1.44 (1.31, 1.59)***	1.49 (1.30, 1.70)***
31–145 DDD	367/897	1.42 (1.24, 1.63)***	1.66 (1.38, 1.99)***
>145 DDD	364/879	1.45 (1.27, 1.66)***	1.79 (1.50, 2.15)***
*P* for trend			<.001

^†^Adjusted for statins, anti-diabetics, NSAIDS, antiarrhythmic drugs of propafenone, quinidine, comorbidities of diabetes, chronic liver disease, cirrhosis, HBV infection, HCV infection, and alcoholism

*P<.05

**P<.01

***P<.001.

**Table 5:** Odds Ratios (ORs) for MNLIHD between Amiodarone and non- Amiodarone group.

	**Non-MNLIHD**		**MNLIHD**			
**Time for Amiodarone use**	**Amiodarone No.**	**%**	**Amiodarone No.**	**%**	**OR^†^**	**(95% CI)**
No use	17437	95.2	8499	91.6	1.00	(reference)
Within one year prior to index	872	4.76	783	8.44	2.34	(2.05, 2.67)***
Within two year prior to index	978	5.31	564	6.22	1.22	(1.06, 1.42)**

^†^Adjusted for statins, anti-diabetics, NSAIDS, antiarrhythmic drugs of propafenone, quinidine, comorbidities of diabetes, chronic liver disease, cirrhosis, HBV infection, HCV infection, and alcoholism

**P<.01

***P<.001.

## Discussion

We conducted a comprehensive population-based case-control study, using NHI databases to investigate the association of antiarrhythmic drug use with the risk of MNLIHD in a group of 29,441 arrhythmia patients. The MNLIHD group (9944 patients) and non- MNLIHD group (19,497 patients) were adjusted for baseline comorbidities, comprising diabetes, chronic liver disease, cirrhosis, HBV infection, HCV infection, and alcoholism, all of which might also be risk factors for MNLIHD. After adjustment for age, sex, statins, anti-diabetics, NSAIDS, propafenone use, and quinidine use, amiodarone users were still more likely to be diagnosed with MNLIHD (OR = 1.60, *P*<.001). The risk of developing MNLIHD was increased further for amiodarone users exhibiting comorbidities (adjusted OR = 18.0, *P*<.001) compared with those without comorbidities (OR = 2.43, *P* <.001), and the risk was also higher for patients who did not use amiodarone but exhibited comorbidities (OR = 12.6, *P* <.001). Among HBV and HCV patients, amiodarone also markedly exhibited an significant increment of MNLIHD risk. In addition, we observed a dose-dependent effect for the amiodarone-associated risk of MNLIHD, whereby patients with an annual mean DDD of ≤30 exhibited an OR of 1.49, those with an annual mean DDD of 31–145 exhibited an OR of 1.66, and those with an annual mean DDD of >145 exhibited an OR of 1.79 (all *P* <.001).

Oral antiarrhythmic agents are often classified according to their molecular targets, which are sodium channels (Class I), β-adrenergic receptors (Class II), potassium channels (Class III), and calcium channels (Class IV). Antiarrhythmics used in Taiwan include amiodarone, mexiletine, propafenone, quinidine, and procainamide, all of which have shared, multiple aforementioned targets [2, 15]. Although few case reports have described liver toxicity arising from the use of some antiarrhythmics, amiodarone has been extensively studied and has been demonstrated to induce chronic liver injury and cirrhosis in patient subgroups [5, 8, 9]. Primary HCC is the fifth leading cause of cancer deaths worldwide, and the second leading cause of cancer deaths in Taiwan [13, 16]. People diagnosed with cirrhosis are at particular risk of developing HCC [17]. Based on our results without adjustment for baseline comorbidities, treatment with amiodarone, propafenone, and quinidine, were all significantly associated with MNLIHD (*P* <.001); however, after adjusting for age, sex, statins, anti-diabetics, NSAIDS, propafenone use, quinidine use, and comorbidities, amiodarone use was the only significant risk factor for MNLIHD among the aforementioned antiarrhythmic drugs (OR = 1.60, *P* <.001).

Su et al. conducted a population-based cohort study to assess the risk of various cancers, including those of the lung, thyroid, skin, and liver, among patients treated with amiodarone, and revealed a borderline significant (standardized incidence ratios, 1.12; 95% CI, 0.99–1.26) increased risk associated with the use of this drug [12]. An analysis of data from a postmarketing surveillance report of the U.S. Food and Drug Administration (FDA) suggested that treatment with amiodarone was associated with the development of lung masses, thyroid cancer, and skin cancer [18]. However, the present population-based, case-control study is the first to report a significant association between the administration of amiodarone and the development of MNLIHD.

Amiodarone, a benzofuran Class III antiarrhythmic agent, was approved by the U.S. FDA in 1985 for the treatment of a wide spectrum of ventricular and supraventricular tachyarrhythmias [2]. Because of its fat-soluble properties and long half-life (53 d), amiodarone can accumulate in the soft tissues during long-term treatment and can thus potentially cause toxicity in several organ systems, including the thyroid gland, lung, skin, and liver [19]. Amiodarone and its N-dealkylated metabolites represent hepatic mitochondrial toxins [20], that can uncouple oxidative phosphorylation, inhibit complex I enzymatic reactions of the electron transport chain, and disrupt fatty acid β-oxidation, all of which are essential biological processes [21–24]. Long-term treatment with amiodarone lasting months or years might cause steatosis. It can evolve into steatohepatitis, a medley of inflammatory process, hepatocellular injury, and fibrosis, often resulting in cirrhosis and HCC [24, 25]. Although previous animal mutagenicity studies have revealed no amiodarone-induced carcinogenicity [26], several case reports have suggested a possible link between amiodarone use and the progression of malignancies in humans [27–32]. Additionally, the structure of amiodarone is similar to that of thyroxine, increasing the risk of both hypothyroidism and hyperthyroidism [33], and patients with thyrotoxicosis and goiter have been reported to have a 3% to 12% increased risk of developing thyroid cancer [34, 35]. In a meta analysis, 4 of 15 randomized controlled trials reported cancer-related deaths and revealed a significant increase in cancer mortality among patients assigned to an amiodarone group compared with a control group (0.8% vs 0.3%) [36]. Similarly, we observed that the presence of MNLIHD was significantly associated with amiodarone treatment, but not with administration of mexiletine, propafenone, quinidine, or procainamide (after adjusting for comorbidities). The lack of association between the administration of these other drugs and MNLIHD might be attributable to the small number of cases and the consequent lack of sufficient statistical power.

Numerous case reports have described the development of cancer following the regular use of amiodarone for 2 to 5 years [32, 37]. It has therefore been suggested that a latency period and the cumulative dose effects of amiodarone might be critical factors in the progression of amiodarone-associated malignancies. This hypothesis was supported by the study of Su et al. [12], in which a significant association was observed among male patients and those with a cumulative DDD (cDDD) of >180 within the first year, and male patients with a cDDD of >180 exhibited a high standardized incidence ratio of 1.46 (*P* = .008). By using the multivariate Cox regression model, they also confirmed a dose-dependent effect in the high and low cDDD tertiles regarding the incidence of cancer, with an adjusted hazard ratio of 1.98 (*P* = .006) [12]. In our study, the risk of MNLIHD was observed to be significantly associated with the dose of amiodarone in the multivariate analysis, after adjusting for age, sex, several medications (statins, anti-diabetics, NSAIDS, propafenone, quinidine) and comorbidities.

This study had limitations; first, the NHIRD in Taiwan does not provide patient details that might have constituted risk factors for MNLIHD, including body mass index, lifestyle factors (eg, physical activity, smoking, and alcohol consumption), environmental exposure, and family history of malignancy, and thus, these were not available for analysis. Second, we were unable to contact the patients directly to obtain additional information because of the anonymized nature of this database. The claims data in the NHIRD are also used primarily for administrative billing purposes and are not analyzed for scientific reliability for clinical studies.

Despite these limitations, this study is the first nationwide population-based case-control study of the association between MNLIHD and the use of antiarrhythmic agents. We observed a higher risk of MNLIHD among patients administered with amiodarone, particularly if they received a high DDD of this drug or exhibited comorbidities. This observed dose-dependent effect is consistent with the findings of Su et al. [12] The present study only addressed the amiodarone-related risk of MNLIHD, and larger scale trials and observational studies are required to assess the risk of amiodarone therapy for a wide range of other cancers.
