# Correction: Antiarrhythmic Agents and the Risk of Malignant Neoplasm of Liver and Intrahepatic Bile Ducts

**DOI:** 10.1371/journal.pone.0121942

**Published:** 2015-03-27

**Authors:** The PLOS ONE Staff

The following information is missing from the Funding section.

This study was supported by grants from the National Science Council, Executive Yuan, Taiwan, ROC (NSC-101-2320-B-039-007-MY3), China Medical University Hospital, Taichung, Taiwan (DMR-103-049), partially supported by the Taiwan Ministry of Health and Welfare Clinical Trial and Research Center of Excellence (MOHW103-TDU-B-212-113002), Health and welfare surcharge of tobacco products, China Medical University Hospital Cancer Research Center of Excellence (MOHW104-TD-B-111-03, Taiwan), and by China Medical University under the Aim for the Top University Plan of the Ministry of Education, Taiwan.
